# Supplementary material for: Copy Number Variant Duplications Associated with Essential Tremor
Source: Tremor Other Hyperkinet Mov (N Y). 2026 Feb 10;16:10. doi: 10.5334/tohm.1132 (PMC12904121; doi:10.5334/tohm.1132)
Supplement: Supplementary File. — Supplemental Figures and Tables. [file tohm-16-1-1132-s1.pdf]

Supplemental Figures and Tables:

ET patients and control individuals underwent quality control at the sample level and CNV level. The number of individuals passing each quality control parameter is shown in Table S1.

Quality Control Parameter	ET Samples	Control Samples
Raw Data	1,853	10,336
European	1,572	9,860
Unrelated	1,507	9,630
Raw CNVs	1,485	9,629
Log R Ratio Standard Deviation	1,261	9,582
B Allele Frequency Standard Deviation	1,242	9,549
Waviness Factor	1,204	9,549
Fragmented Call Correction	1,204	9,549
Size (CNVs > 30kbs)	1,150	8,132
CNV Number per Sample	1,092	7,974
Quality Score	618	6,485
Rare	572	6,155
Blacklist	536	5,828
Consensus	505	5,563
DeepCNV	407	4,870
Protein Coding	244	2,402

Table S1: The number of ET and control individuals remaining after each quality control step. Rare CNVs were defined by Gnomad V4 structural variant European allele frequencies < 1%. Consensus CNVs were CNVs that were called by both PennCNV and QuantiSNP algorithms.

The exact number of deletions and duplications which remain after each quality control step for ET cases and controls is shown is Table S2:

Quality Control Parameter	Essential Tremor Patients		Controls	
	Deletions Count	Duplications Count	Deletions Count	Duplications Count
Raw CNVs	106,118	7,992	140,818	42,104
Log R Ratio Standard Deviation	50,508	7,070	133,247	42,360
B Allele Frequency Standard Deviation	49,833	7,036	131,109	42,253
Waviness Factor	42,328	6,197	130,404	41,823
Fragmented Call Correction	41,860	6,112	127,377	39,842
Size	16,021	3,688	27,401	14,207
CNV Number per Sample	9,011	3,393	15,483	13,473
Quality Score	701	419	8,399	5,635
Rare	618	336	7,348	4,783
Blacklist	576	280	7,031	3,937
Consensus	563	230	6,794	3,247
DeepCNV	405	207	5,096	2,842
Protein Coding	143	180	1,348	1,709

Table S2: The number of total deletions and duplications which remain after each quality control step for ET cases and controls. Rare CNVs were defined by Gnomad V4 structural variant European allele frequencies < 1%. Consensus CNVs were CNVs that were called by both PennCNV and QuantiSNP algorithms.

All rare CNVs mapped to protein coding regions that were retained for analysis are shown in Figure S1 based on chromosome and position, colored by disease status and CNV type.

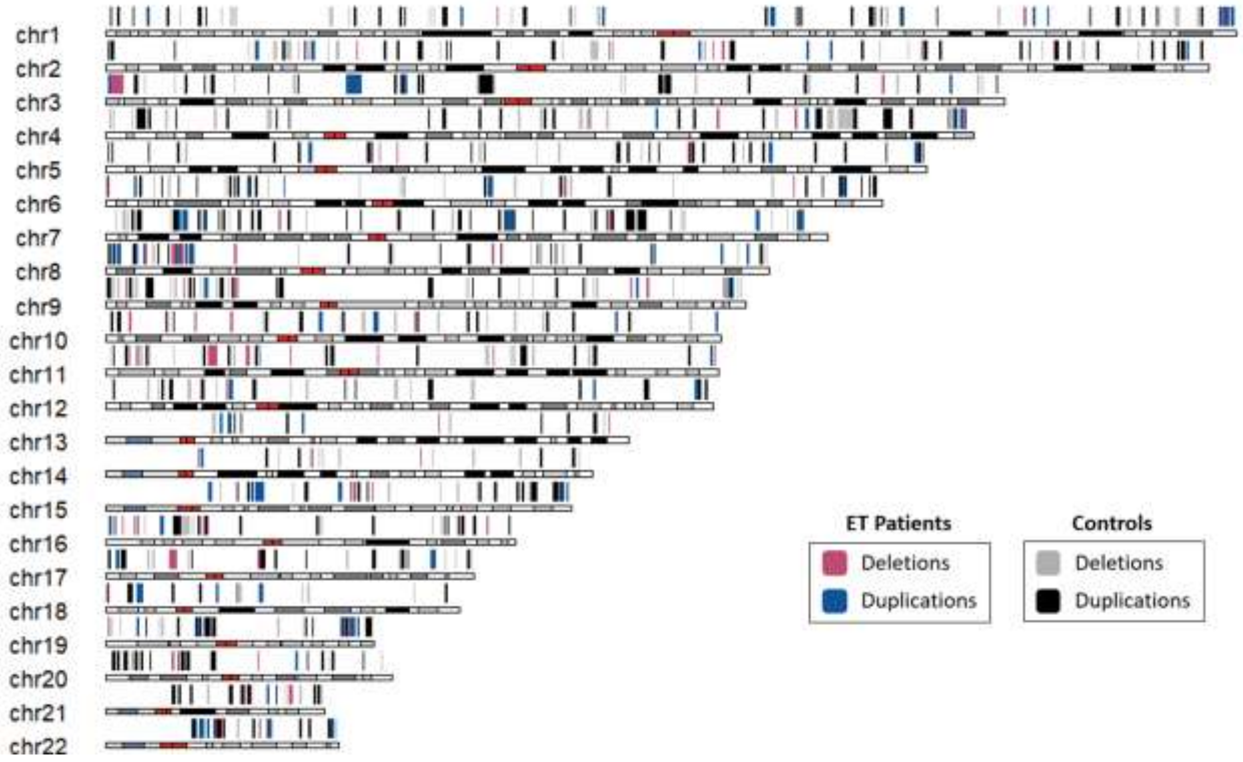

Figure S1: Rare copy number variants mapping to protein coding regions shown across the genome. Deletions and duplication are shown above the locus they map to per chromosome for ET patients and control individuals. ET patient deletions are shown by red boxes while ET duplications are shown by blue boxes. Deletions in the controls are shown by gray boxes while duplications in controls are shown by black boxes.

The ancestry of samples was inferred by principal component analysis (PCA) using 1000G as reference. The first two genetic principal components (PCs) are shown in Figure S2.

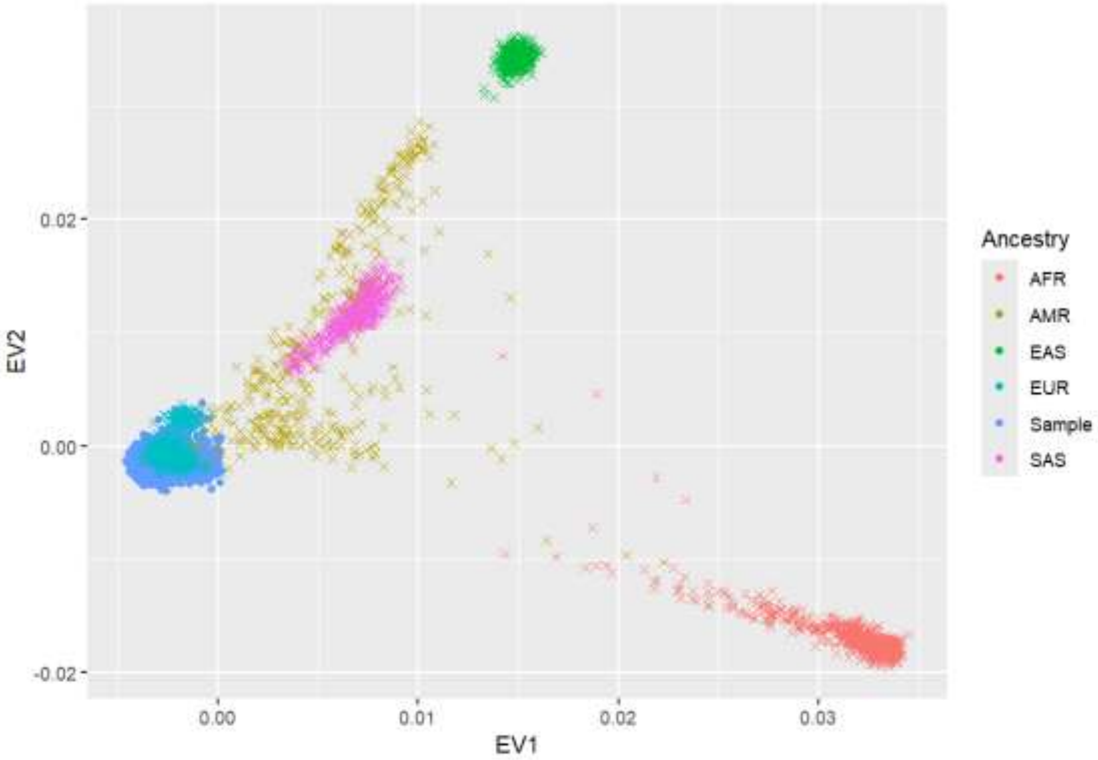

Figure S2: Principal component analysis to infer sample ancestry. Sample ancestry was estimated using principal component analysis (PCA) using PLINK v.1.9, with the 1000 Genomes Project as a reference panel. As 94% of samples clustered with European samples of the 1000 Genomes Project, only individuals with inferred European ancestry were included in the analysis. EV1 represents the first principal component while EV2 represents the second principal component.

The Scree plot capturing variance per principal component of the ancestry PCA is shown in Figure S3.

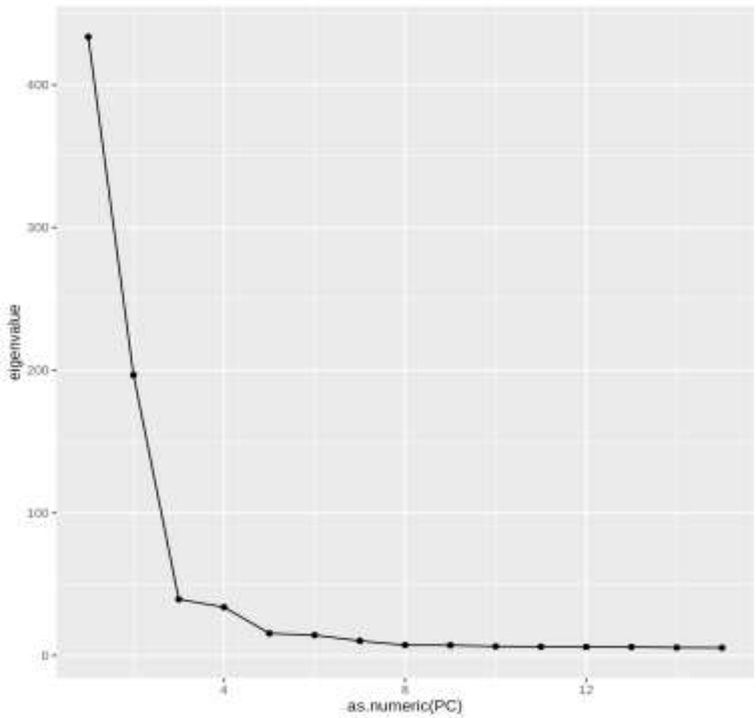

Figure S3: Scree plot showing the variance explained by each principal component in the ancestry PCA analysis. The x-axis represents the principal component number (PC) while the y-axis shows the eigenvalue which captures the variance explained by each PC.

A figure showing the clustering of the cases and controls coloured separately across the first and second genetic principal components was generated to demonstrate how genetically similar the samples (cases and controls) are as seen by their genetic ancestry overlap. The first two principal components capture the largest genetic differences between samples which represents ancestry. Cases and controls are coloured differently in blue and purple respectively in Figure S4.

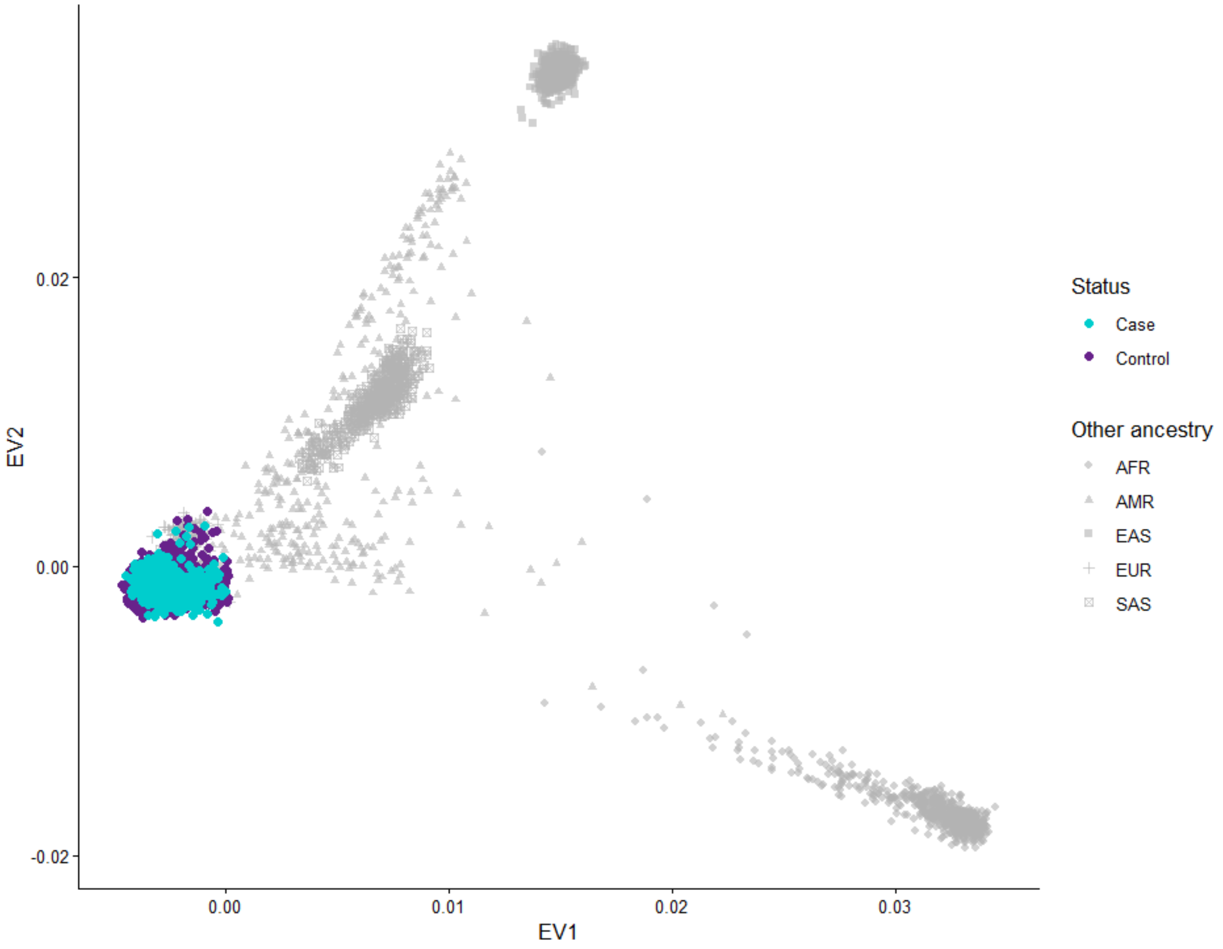

Figure S4: Principal component analysis showing case and control ancestry. Sample ancestry was estimated using principal component analysis (PCA) using PLINK v.1.9, with the 1000 Genomes Project as a reference panel. Only samples of genetic European ancestry were retained following sample level quality control. EV1 represents the first principal component while EV2 represents the second principal component. All ancestry references are shown in gray with different shapes showing different ancestries. Cases are shown in blue while controls are in purple. The overlap between cases and controls show samples surviving sample level quality control are genetically similar and overlap with European reference samples.

A QQ-plot for gene-wise burden testing across deletions and duplication events was generated in R using the qqman() package shown in Figure S5. Notably, since we are testing the number of rare CNV events affecting a given gene, Fisher's exact test contingency tables are comprised of rather small counts which ultimately produce a limited set of possible p-values. This is reflected in the plateauing effect seen in Figure S5 as fewer tests (affected genes) and counts (number of CNVs) appear in rare event testing leading to limited, and thus several repeating, test statistic outcomes.

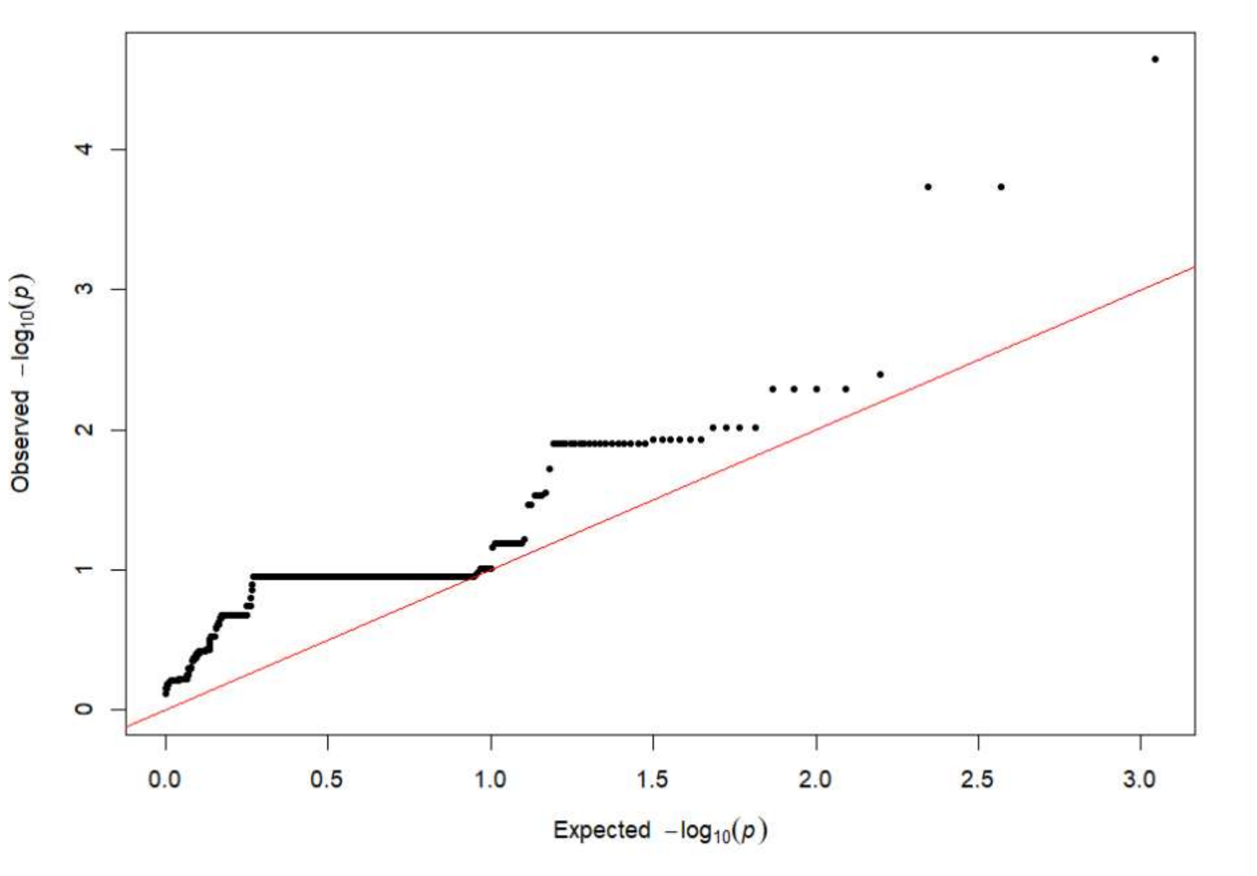

Figure S5: QQ-plot of global gene-wise burden across deletions and duplications. The diagonal red line illustrates the expected distribution under the null hypothesis. The plateauing of p-values reflects the limited resolution of tests with small CNV counts due to the focus on rare events.
